# Developing a conceptual framework for implementation science to evaluate a nutrition intervention scaled-up in a real-world setting

**DOI:** 10.1017/S1368980019004415

**Published:** 2021-04

**Authors:** Haribondhu Sarma, Catherine D’Este, Tahmeed Ahmed, Thomas J Bossert, Cathy Banwell

**Affiliations:** 1 Research School of Population Health, Colleague of Health and Medicine, The Australian National University, Canberra, ACT 2601, Australia; 2 Nutrition and Clinical Services Division, icddr,b, Dhaka 1212, Bangladesh; 3 School of Medicine and Public Health, Faculty of Health and Medicine, The University of Newcastle, Callaghan, NSW 2308, Australia; 4 Department of Global Health and Population, Harvard T.H. Chan School of Public Health, Boston, MA, USA

**Keywords:** Implementation science, Nutrition implementation science, Conceptual framework, Implementation of intervention

## Abstract

**Objective:**

The aim of this paper is to identify and develop a comprehensive conceptual framework using implementation science that can be applied to assess a nutrition intervention in a real-world setting.

**Design:**

We conducted a narrative review using electronic databases and a manual search to identify implementation science frameworks, models and theories published in peer-reviewed journals. We performed a qualitative thematic analysis of these publications to generate a framework that could be applied to nutrition implementation science.

**Results:**

Based on this review, we developed a comprehensive framework which we have conceptualised as an implementation science process that describes the transition from the use of scientific evidence through to scaling-up with the aim of making an intervention sustainable. The framework consisted of three domains: Domain i – efficacy to effectiveness trials, Domain ii – scaling-up and Domain iii – sustainability. These three domains encompass five components: identifying an ‘effective’ intervention; scaling-up and implementation fidelity; course corrections during implementation; promoting sustainability of interventions and consideration of a comprehensive methodological paradigm to identify ‘effective’ interventions and to assess the process and outcome indicators of implementation. The framework was successfully applied to a nutrition implementation program in Bangladesh.

**Conclusions:**

Our conceptual framework built from an implantation science perspective offers a comprehensive approach supported by a foundational and holistic understanding of its key components. This framework provides guidance for implementation researchers, policy-makers and programme managers to identify and review an effective intervention, to scale it up and to sustain it over time.

Implementation science is becoming increasingly important in the nutrition field. Over the past decades, a number of innovative nutrition interventions have been developed and tested that can contribute to reduce the high burden of malnutrition and human development, particularly when they are scaled-up through the use of a comprehensive implementation science framework. Globally, about 2 billion people are suffering from anaemia mainly due to micronutrient malnutrition^(1)^. A high burden of malnutrition imposes adverse consequences on overall development, which impacts on individuals, families, communities and nations. Globally, it is estimated that all forms of malnutrition cost up to US$3·5 trillion per year^(2)^.

Recently, several initiatives have been taken at the global level to address all forms of malnutrition including locating nutrition targets within the sustainable development goals (SDG). The SDG 2 goals aim to end hunger, achieve food security and improve nutrition and promote sustainable agriculture by 2030^(3)^. Another initiative by the Scaling-Up Nutrition Movement focuses on translating momentum into results for people who suffer due to malnutrition^(4)^. The application of implementation science to nutrition is important to expedite the use of effective nutrition interventions. Over the last couple of decades, the number of randomised controlled trials (RCTs) has increased five to tenfold to test the efficacy of innovative health and nutrition interventions^(5)^, although there is little evidence that these trials transfer into real-world practice^(6)^.

Implementation researchers have proposed a number of conceptual frameworks and theories which have diverse implications and usefulness for a systematic uptake of evidence-based interventions into the complex real world. These theories, models and frameworks have been categorised into three groups^(7)^: (i) process models – guiding the process of translating research into practice; (ii) determinants frameworks, classic theories and implementation theories – explaining what influences implementation outcomes and (iii) evaluation frameworks evaluating the implementation^(7)^. However, the scaling-up and sustainability of an implementation have not been included in these groups, even though they are essential components of implementation science^(8,9)^. Scaling-up and sustainability focus on specific processes that help expand the impact of innovative interventions across populations to ensure continuation of the impact for a longer term or until the problem is resolved.

Recently in the nutrition field, several scholars have used implementation science to support scaling-up of evidence-based nutrition interventions^(10–^ ^12^ ^)^. In 2016, the Society for Implementation Science in Nutrition (SISN) was formed to promote implementation science for addressing malnutrition burdens in low- and middle-income countries^(13)^. In 2018, researchers in SISN defined implementation science in nutrition, ‘as an interdisciplinary body of theory, knowledge, frameworks, tools and approaches whose purpose is to strengthen implementation quality and impact’^(12)^. The same article also proposed an integrated framework for implementation science in nutrition^(12)^. This framework addressed several elements of implementation science including recognising the quality of an implementation, developing a culture of evaluation and learning among program implementers and resolving inherent tensions between program implementation and research^(12)^. These initiatives may be further strengthened by developing a comprehensive conceptual framework for nutrition implementation science that includes how an effective intervention can be identified for scaling-up and sustained until malnutrition is improved at the population level.

The aim of this paper is to develop a more comprehensive conceptual framework that can be applied to a real-world nutrition intervention from an efficacy or effectiveness trial through scaling-up to its sustainability. This work was motivated by the need to implement a package of nutrition interventions under the platform of Maternal, Infant and Young Child Nutrition in a low-income setting. The first author of this paper developed this framework to evaluate the implementation of home fortification of food with micronutrient powder (MNP) in Bangladesh.

## Methods

### Search strategy

To perform a narrative review of peer-reviewed published literature on implementation science frameworks or theory, we used PubMed and Scopus databases, and Google and Google Scholar as search engines and performed manual searches (e.g. reference searching, personal communication) to identify relevant literature and documents. The final round of searching finished on 31 December 2018, and we did not limit publication date while searching.

#### Functional definitions

We used a range of key terms and terminologies in this paper, including Narrative review, Scaling-up, Efficacy, Effectiveness, Fidelity, Concurrent evaluation, Sustainability, Implementation science and Conceptual framework. Table 1 shows the functional definitions of these terms/terminologies for this paper.

**Table 1:** Definition of key terminologies

Key terminologies	Definitions
Narrative review	A narrative review systematically identifies and summarises narrative evidence in the literature to address a predefined research question. It considered new research question and looks for new study areas not yet addressed^(14)^
Scaling-up	An initiative to increase the coverage of an effective intervention across a larger population setting
Efficacy	The ability of an intervention to produce the anticipated positive outcome to address a defined health problem
Effectiveness	When an intervention is shown to have proven efficacy in a real-world setting. Effectiveness indicates quality, outcomes, benefits, harms and appropriateness of an intervention and helps policy-makers decide if an intervention should be scaled-up
Fidelity	It means to implement an intervention as it was originally planned. In this paper, we further argue that the fidelity of an intervention improves at scaling-up after adapting with contextual factors of the setting during implementation
Concurrent evaluation	A flexible, mixed-method evaluation – implemented at the same time and alongside the implementation of the programme implementation. Concurrent evaluation particularly to assess continuously the progress of a particular program in a particular community, thereby determining how a program works and with whom it works; and, accordingly, to make necessary corrections^(15)^
Sustainability	Continuation of programme activities, outcomes and impact over a period of time or as long as needed to solve the health problem
Implementation science	Implementation science is a systematic study to identify an ‘effective’ intervention for scaling-up in a real-world setting, consider real-time evaluation and a feedback-loop mechanism to improve implementation and effectiveness for sustaining the intervention and its outcome over the long term. Other definitions of implementation science focused on the process and fidelity of implementation by emphasising the integration and evidence-based uptake and transfer of an intervention into routine healthcare systems^(8–^ ^10^ ^,^ ^16^ ^–^ ^21^ ^)^. The previous definitions did not consider a real-time evaluation for supporting a timely feedback loop and sustainability of implementation, whereas these are important components of implementation science
Conceptual framework	‘Conceptual framework’ is a visual or written product, which displays either a partial or a complete pathway from intervention innovation to practice in a real-world setting. It explained either graphically or in narrative form, the main things to be studied – the key factors, concepts or variables – and the presumed relationships among them^(22)^. The conceptual framework sets the stage for the presentation of the particular research question that drives the investigation being reported based on the problem statement^(23)^

### Search terms

For PubMed, we used (‘implementation science’[Title/Abstract] OR ‘implementation research’[Title/Abstract]) AND (‘framework’[Title/Abstract] OR ‘theory’[Title/Abstract] OR ‘model’[Title/Abstract]) AND English[lang] as search terms and search limit. For Scopus, we used [(TITLE-ABS-KEY (‘implementation science’) OR TITLE-ABS-KEY (‘implementation research’) AND TITLE-ABS-KEY (‘framework’) OR TITLE-ABS-KEY (‘theory’) OR TITLE-ABS-KEY (‘model’)) AND (LIMIT-TO (SUBJAREA, ‘MEDI’) OR LIMIT-TO (SUBJAREA, ‘SOCI’) OR LIMIT-TO (SUBJAREA, ‘PSYC’) OR LIMIT-TO (SUBJAREA, ‘NURS’)) AND (EXCLUDE (SUBJAREA, ‘BUSI’) OR EXCLUDE (SUBJAREA, ‘COMP’) AND (LIMIT-TO (LANGUAGE, ‘English’)) as search terms and search limit. However, when we used Google and Google Scholar, we used combination of these search terms or used the title of the article.

### Exclusion criteria

We excluded literature that was not relevant to the objectives of this paper; for example, if it did not propose a unique framework, but instead critically appraised existing frameworks. We also excluded literature that dealt with the same framework in multiple papers. In this case, we included the paper that proposed the original framework or the paper that modified the framework later.

### The process of selecting literature

We used EndNote version X8.2 for organising and managing the literature review. First, we removed the duplicate articles identified through both the search databases: Scopus and PubMed. Then we screened the titles of all identified articles and excluded those which were not relevant to the study objective. We read the abstracts of all remaining articles and selected the literature which contained a framework, model or theory pertaining to implementation science. We downloaded full texts of the selected articles and excluded the articles if full texts were not available. We read the full texts of the selected articles and excluded those which fallen under the exclusion criteria. We included and reviewed articles identified in the earlier full-text review to produce the final list of articles discussed below.

### Analysis and synthesis to identify the components of conceptual framework

We performed a qualitative thematic analysis on the identified articles containing implementation science frameworks. We reviewed the articles, highlighting the text that explained the frameworks and then coded the text to build initial themes. At first, we performed a matrix analysis to display the reviewed findings. We later grouped initial themes under five major themes, then summarised them in tabulation forms (Tables 2 and 3). This process helped us to identify which specific components of implementation science have been included in the reviewed articles. We have summarised the reviewed findings and used them to develop a comprehensive conceptual framework with additional evidence from the literature to clarify the domains and components of the framework.

**Table 2:** A summary of the literature review

Authorar [reference #]	Objective	Discipline	Components addressed				
			Effective intervention	Implementation fidelity	Concurrent evaluation	Course corrected during implementation	Sustainability
Tumilowicz *et al*.^(10)^	Presents an approach to implementation science in nutrition	Nutrition		X	X	X	
Menon *et al*.^(11)^	To describe a nutrition implementation-focused framework and research agenda	Nutrition		X	X		X
Gillespie^(12)^	To synthesise what is known about nutrition to guide actions that focus on scaling-up impact on nutrition	Nutrition	X	X	X		
Damschroder *et al*.^(24)^	Describe the Consolidated Framework for Implementation Research (CFIR)	Health service	X	X			
Kitson *et al*.^(25)^	Support successful implementation of research into practice	Nursing	X	X	X		
Glasgow *et al*.^(26)^	To propose a model for evaluating the public health interventions	Public health	X	X	X		X
Carroll *et al*.^(27)^	To understand whether an intervention has been implemented with fidelity, how and why an intervention works and the extent to which outcomes can be improved	Public/Mental health		X	X		
Kilbourne *et al*.^(28)^	Describe the use of a conceptual framework and implementation protocol to prepare effective health services interventions	Health service	X	X			X
Van Achterberg *et al*.^(29)^	To provide an introduction and overview of current developments in implementation science and to apply these to nursing	Nursing	X	X	X		
Scheirer *et al*.^(30)^	To provide guidance for research and evaluation of health program sustainability	Public health					X
Fassier *et al*.^(31)^	To construct a conceptual framework to identify barriers and facilitators before implementing and to validate this conceptual framework empirically	Occupational health		X			
Rongey *et al*.^(32)^	To illustrate how this hybrid model could inform the translation of a novel method of healthcare delivery	Health service		X			
Knapp *et al*.^(33)^	To integrate relevant elements of implementation science theories for conducting real-world implementation	Health care	X	X		X	X
Meyers *et al*.^(34)^	To the understanding of the complex and dynamic nature of implementation	Psychology	X	X	X	X	
Chambers *et al*.^(35)^	Understandings dynamic approach to sustainability and implications of this framework for research, policy and practice	Health care		X			X
Flottorp *et al*.^(36)^	Describe the development of a comprehensive, integrated checklist of determinants of practice	Health care		X			
Lobb *et al*.^(37)^	Provides a review of implementation science to outlined several ways in which implementation is being applied to population health	Population health		X			
Sivaram *et al*.^(38)^	Describe the challenges of implementing cancer control programs and present a framework to illustrate how it can be applied in the context of global cancer research and practice	Health service	X	X	X		
Neta *et al*.^(39)^	To present and discuss a framework for enhancing the value of research for dissemination and implementation of improving population health	Population health		X	X		**X**
Atkins *et al*.^(40)^	Describe a process that aligns ecological theory with a public health model to address long-standing mental health disparities	Public health		X			X
Barker *et al*.^(41)^	Describe a framework for taking health interventions to full scale, and use two large-scale improvement initiatives in Africa to illustrate the framework in action	Health service		X			X
Pérez *et al*.^(42)^	Propose a modified framework for implementation fidelity to provide a better fit for adaptive interventions	Public health		X			
Blanchard *et al*.^(43)^	Describes active implementation frameworks to facilitate the implementation and improvement of comprehensive medication management	Health service	X	X		X	
Pfadenhauer *et al*. ^(44)^	To develop a framework to assess context and implementation of complex interventions	Public health		X			
Rapport *et al*.^(45)^	To reveal how implementation science is presented and understood in health services research contexts and clarify the foundational concepts	Health service		X	X	X	X
Vanderkruik *et al*.^(46)^	Present a framework for evaluating contextual factors affecting an initiative at multiple phases of its life cycle	Public health		X	X		
Shediac-Rizkallah *et al*.^(47)^	To know what factors influence sustainability, provide strategy to fostering program sustainability and provide a future direction	Public health					X
Schell *et al*.^(48)^	To present a new conceptual framework for program sustainability in public health	Public health					X
Iwelunmor^(49)^	To conduct a systematic review of empirical literature to explore how health interventions implemented in Sub-Saharan Africa are sustained	Public health					X
Verhagen *et al*.^(50)^	To bridge the gap between knowledge derived from research and generate evidence-based usable information and tools for practice	Sport science	X	X	X		

**Table 3:** Synthesis of review findings

Component	Review findings
Identifying an effective intervention	Understand what exactly is to be scaled-up to achieve large-scale impact^(12)^ Intervention characteristics: intervention sources, evidence strength and quality, relative advantage, adaptability, trialability, complexity, design quality and packaging^(24)^ Assess the nature and strength of the evidence and its potential for implementation^(25)^ Determining the impact of intervention through assessing reach and efficacy of an intervention implemented in a real-world setting by individuals who are not part of the original research^(26)^ Pre-conditions – identifying need, target population and suitable intervention and pre-implementation – intervention packaging and community input^(28)^ The effective implementation starts with the identification of relevant practice issues (problems or best practices) and matching research findings or guidelines^(29)^ Explore what needed – conducting a comprehensive formative evaluation to determine the nature of what service or resource is needed^(33)^ Initial consideration regarding the host setting, conduct need assessment and readiness assessment^(34)^ Gather evidence that a technology or modality works from basic science as well as controlled assessments of effectiveness^(38)^ Exploration – assess fit to ensure a useable innovation^(43)^ Evidence synthesis and describe the problem as encountered in practice in terms of problem magnitude, severity, societal burden and problem context^(50)^
Implementation fidelity	Assess implementation process to understand the quality of implementation^(10)^ Provided a pragmatic and high-level summary of the implementation factors and processes^(11)^ Focusing on scaling-up strategy, process and pathways (need to address context, process, enabler and barriers of scale-up)^(12)^ Outer setting, inner setting, characteristics of the individuals involved and the process of implementation^(24)^ Addressing context or setting’s characteristics for successful implementation^(25)^ Assessing adoption process of and barriers to adaption for a successful implementation of intervention^(28)^ Measuring implementation fidelity means evaluating whether the result of the implementation process is an effective realisation of the intervention as planned by its designers^(27)^ Implementation – package dissemination, training, technical assistance and evaluation^(28)^ The importance of building bridges between the innovation and the context^(29)^ Understand barriers and facilitators at individual, organisation, legal and political level before implementing an intervention^(31)^ Gave importance of contextual factors and consider patient and community variables such as geography (e.g. distance to care)^(32)^ Deliverable, implementation guide, activities, pre-training and training^(33)^ Create and organise a structure for implementation^(34)^ Consider change that happened over time in the use of intervention, characteristic of settings and ecological systems (policy, population characteristics)^(35)^ Consider determinants of implementation (guideline factors, individual health professional factors, patient factors, professional interactions, incentives and resources, capacity for organisational change, and social, political and legal factors)^(36)^ Consider stakeholder input and partnerships to increase the relevance of research to practice settings and improved public health benefits^(37)^ At the planning phase considered community, individual and social determinants; at guiding programme implementation consider health systems (provider and facilities)^(38)^ Considered evidence-based intervention with programme logic and mechanism of change^(39)^ Considered setting characteristics, implementation strategy and partnership for planning to disseminate and implement an intervention^(39)^ At the implementation stage delivery of intervention to be ensured by reach, adoption and fidelity of implementation^(39)^ Identifying setting, core goals, key opinion leaders and resources for implementation^(40)^ Setup, develop scalable unit and go for full scale-up^(41)^ A comprehensive assessment of the implementation fidelity-adaptation balance^(42)^ Create, implementation team, examine implementation drivers and develop fidelity measure^(43)^ Consider the three dimensions context, implementation and setting^(44)^ Implementation – an ideal and endeavour and the effective implementation stage – the successful endpoint; adoption – the degree of uptake of new ideas, behaviours, practices and organisational structures^(45)^ Inform development and implementation of an initiative^(46)^ Establish a knowledge transfer group consisting of representativeness of key stakeholders, practitioners and researchers with expertise on the evidence at hand^(50)^
Concurrent evaluation	Require a culture of inquiry, evaluation and learning^(10)^ Consider interdisciplinary research to assess coverage, equity, utilisation, demand, outcomes and impacts^(11)^ Monitoring and evaluation, learning, accountability^(12)^ Context or environment into which the research is to be placed, and the method or way in which the process is facilitated^(25)^ Evaluating the interventions to assess positive and negative outcomes as we as public health impact^(26)^ Evaluation must measure all the factors listed above that influence the degree of implementation fidelity, such as intervention complexity and the adequacy of facilitation strategies^(27)^ For effective implementation, requires continuous evaluation and adapting plan^(29)^ Process evaluation to support the implementation effort effectively^(34)^ Conducting programme evaluation to inform policy^(38)^ Consider evolution to assess intervention effectiveness and economic evaluation: cost-benefit/effectiveness budget impact^(39)^ Evaluation frameworks should be in place as part of new research design, ready to be applied at the appropriate stage in the implementation process^(45)^ Evaluation of contextual factors^(46)^ Evaluate within the RE-AIM Framework (reach, effectiveness, adoption, implementation and maintenance)^(50)^
Course correction during implementation	Response among program implementers and resolving inherent tensions between program implementation and research^(10)^ Real-time feedback-loop processes^(33)^ Supportive feedback mechanism – an effective process through which key findings from process data related to implementation are communicated, discussed and acted upon^(34)^ Suggested policy-practice feedback loop – data-driven process to inform decision making around innovation improvement and institutionalisation^(43)^ Consider a feedback loop that demands monitoring, adoption and extended uptake phases, so that with each cycle, the intervention becomes more firmly entrenched within a system^(45)^
Sustainability	The strategic and management capacities for sustainability^(11)^ Long-term maintenance of implementation essential in the collection of programme-level measures of institutionalisation^(26)^ Maintenance and evolution (e.g. preparing the intervention for sustainability)^(28)^ Considered dependent and independent variables for assessing sustainability: dependents variables (benefits and out to be continued; continuation of programme activities, partnership, adapting new organisational practice and sustaining attention to the issue or problem, diffusion and replication in other sites), and independent variables (intervention characteristics, organisational level factors, community-level factors)^(30)^ Sustainability involves the implementation team handing over the continuation of the project the local champion and stakeholders enter the confirmation stage^(33)^ Dynamic view of sustainability – ongoing evidence that can be cycled to continuously improve intervention design, testing and ongoing system change^(35)^ Long-term outcomes: sustainability evaluability transportability replication and uptake of intervention^(39)^ Identify available resources to implement and sustain the service model^(40)^ Adoption mechanism and system support for sustainability^(41)^ Sustainability: once new knowledge and the intervention have been successfully applied and embedded^(45)^ Use of programmatic approaches and strategies that favour long-term program maintenance^(47)^ Considered core domains that affect a program’s capacity for sustainability^(48)^ Framework with factors that influence sustainability – the intersection between the intervention itself and broader socio-cultural and community context within which the intervention is implemented as well as the role of organisational factors in influencing the sustainability of the intervention over time^(49)^

## Results

### Review results

**Fig. 1:**
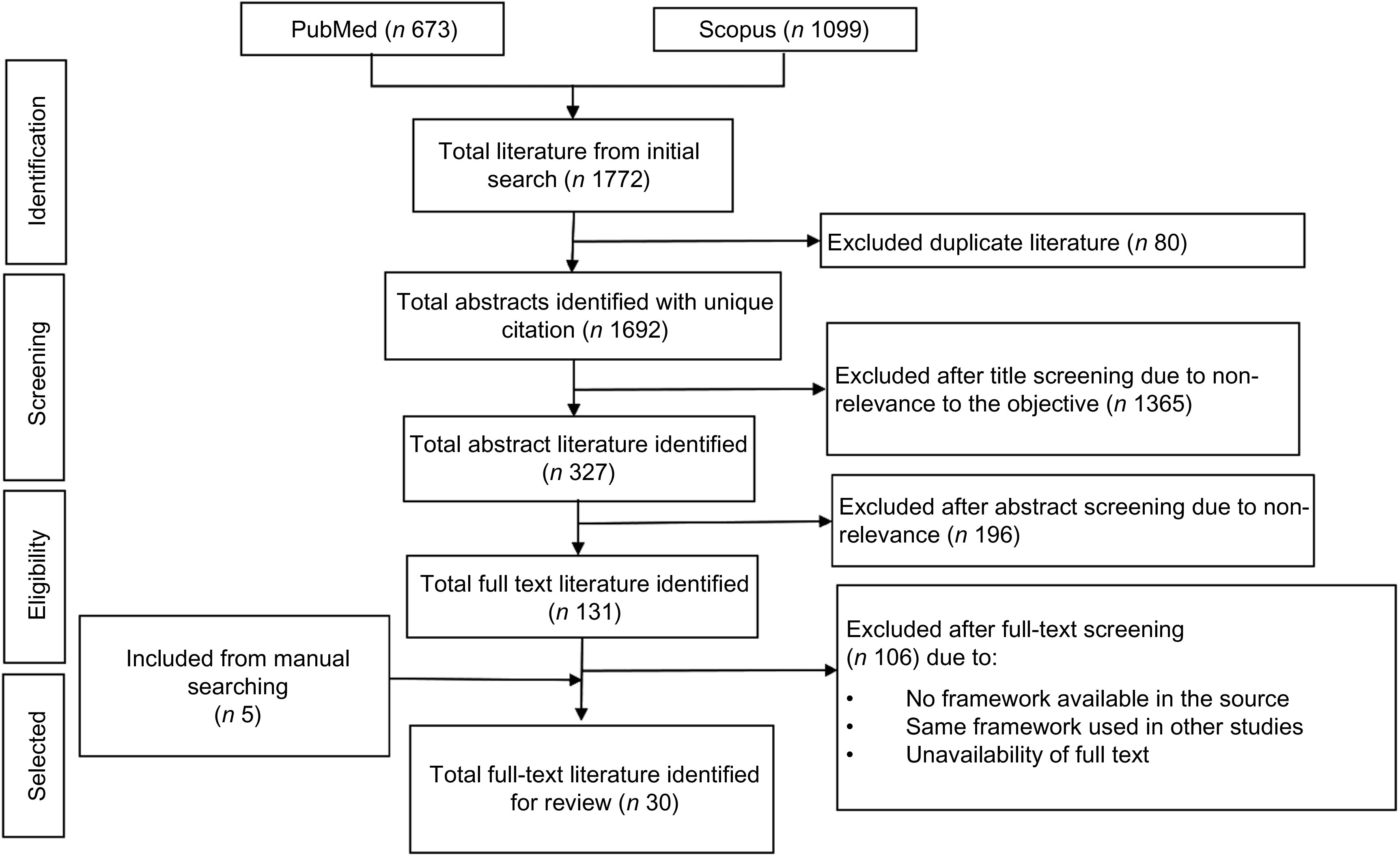
Flow diagram of the literature search and identifying full-text literature

Using both search databases we identified 1772 articles published between 1998, when papers first appeared on implementation science and 2018 when we conducted the search. We excluded 80 duplicate articles. Through title screening, we excluded 1365 articles, which were not relevant to the study’s objectives, and identified 327 articles for abstract screening. We excluded 196 abstracts that were not related to the study objective and four articles due to unavailability of the full text. After reading 127 full-text articles, we excluded 102 because they did not include a framework or they provided duplicates of existing frameworks in the literature. We added five papers through reviewing the references of selected articles. Finally, we identified thirty articles (Fig. 1), which provided a framework, theory or a model for implementation science^(10–^ ^12^ ^,^ ^24^ ^–^ ^50^ ^)^.

Most of the frameworks covered broad public health disciplines, including population health, health services, health care and a few referred to nursing, psychology/mental health, sports science and occupational health. Three frameworks focused on nutrition but did not include all the key components recommended by implementation science. For example, two papers on nutrition implementation science focused on scaling-up a nutrition intervention, but did not consider sustainability in their conceptual frameworks, and the other framework considered sustainability that did not consider how to identify an effective intervention (Table 2).

Table 3 synthesises the key findings of literature review under five broad themes. Eleven frameworks considered how to identify effective interventions^(12,24–^ ^26^ ^,^ ^28^ ^,^ ^29^ ^,^ ^34^ ^,^ ^38^ ^,^ ^43^ ^,^ ^50^ ^)^. The nature of the implementation settings, intervention characteristics, evidence strength and intervention contexts were used to identify effective interventions for scaling-up (Table 3). Most of the frameworks (*n* 26) emphasise the importance of implementation fidelity while scaling-up the intervention in a real-world setting (Table 2). Some frameworks suggested that it was important to identify implementation drivers (e.g. performance of health workers) during scaling-up to measure and improve implementation fidelity. The range of implementation drivers influencing fidelity were found to operate at the health-systems level, the socio-cultural and political level and the individual level. These frameworks also emphasised the importance of guidelines, strategies or structure of the implementation to monitor fidelity, adapting it to local contexts (Table 3).

More than one-third of implementation science frameworks (*n* 13, Table 2) considered real-time evaluation. These evaluations assessed the implementation process, outcome, effectiveness (including cost-effectiveness) and impact of the implementation^(10–^ ^12^ ^,^ ^25^ ^–^ ^27^ ^,^ ^29^ ^,^ ^34^ ^,^ ^38^ ^,^ ^39^ ^,^ ^45^ ^,^ ^46^ ^,^ ^50^ ^)^. It was considered vital that a culture of inquiry, interdisciplinary research and an evaluation framework should be included at the beginning of the implementation strategy (Table 3). Five frameworks included a planned evaluation to improve programme implementation^(10,33,34,43,45)^. Evaluations containing a real-time feedback loop generated key findings. If the data from this was acted upon quickly, it could resolve inherent tensions between program implementation and research (Table 3).

Thirteen frameworks^(11,26,28,30,33,35,40,41,45,47–^ ^49^ ^)^ included sustainability as part of a implementation science framework. The determinants of sustainability covered institutionalisation, adoption mechanisms, system support, involvement of a local champion and consideration of programmatic approaches and strategies (Table 3).

### A comprehensive framework for implementation science

**Fig. 2:**
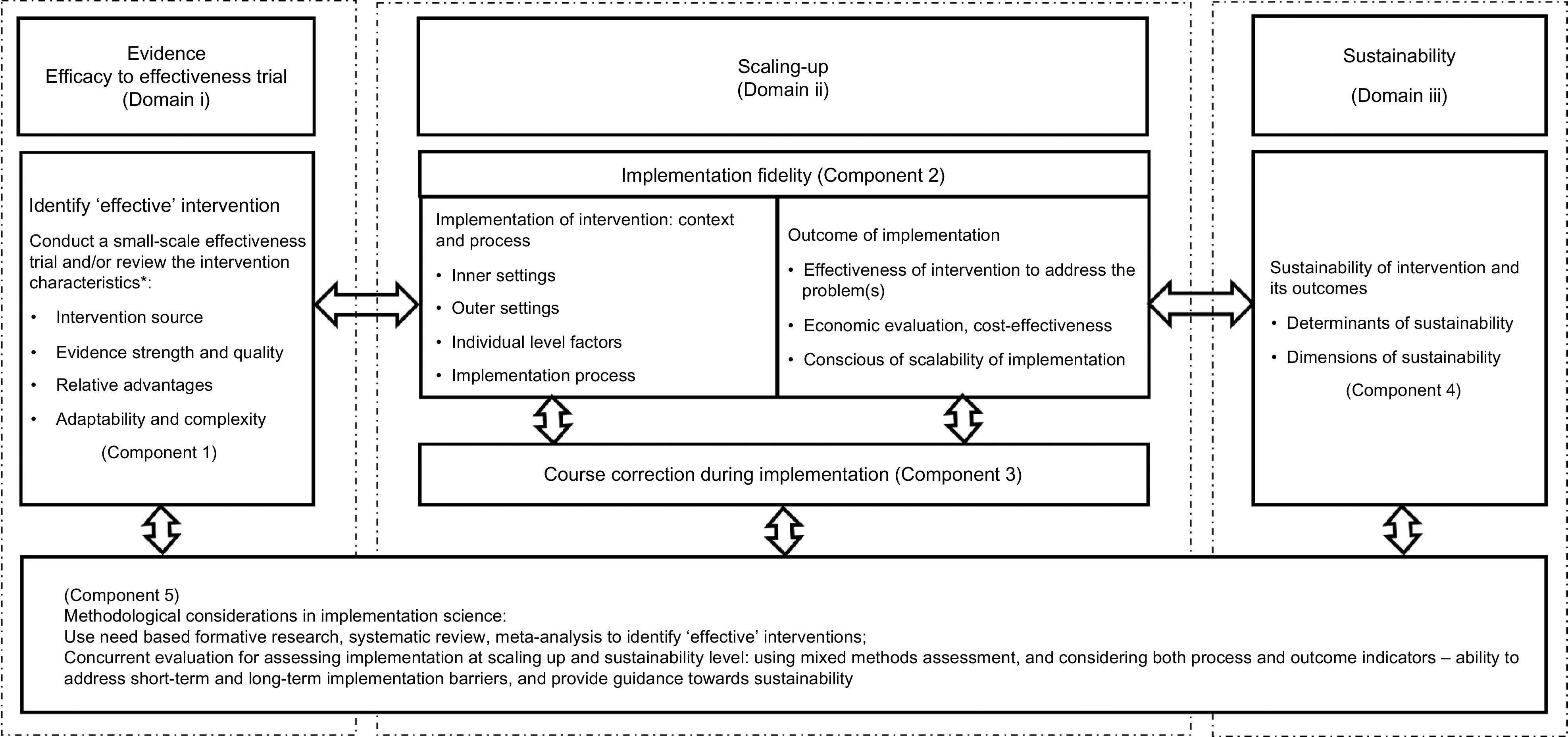
A comprehensive conceptual framework for implementation science

We developed a comprehensive framework which we have conceptualised as an implementation science process that describes the transition from the use of scientific evidence through to scaling-up with the aim of making an intervention sustainable. We grouped the five themes (Tables 2 and 3) under three domains: the Evidence Domain i – efficacy to effectiveness trials, Domain ii – scaling-up and Domain iii – sustainability. (see Fig. 2). The evidence-based intervention should be generated through a scientific process and tested rigorously (such as through an RCT). If the intervention is identified as ‘effective’, it then moves to the phase of scaling-up taking into account the larger setting. When the intervention has demonstrated effectiveness in this larger setting, it has the capacity to be sustainable in a real-world practice setting. The double-headed arrows in the framework indicate the bi-directional interaction between the domains and components (Fig. 2). The process may move from identifying an effective intervention to scaling-up and then to sustainability and in the opposite direction indicating that the intervention or a part of intervention may move back for further assessment or piloting in a small-scale setting. For example, during scaling-up, a researcher may identify an additional intervention characteristic which needs further assessment, or may identify a new problem at scaling-up that requires further experiment.

### Domain i: evidence – efficacy to effectiveness

Domain i deals with how a new evidence-based intervention become an ‘effective’ intervention to qualify for scaling-up in a larger real-world setting.

#### Component 1: identifying an ‘effective’ intervention

Implementation science requires an effective and ‘innovative’ intervention. An intervention can be identified as ‘effective’ through review of the essential intervention characteristics^(50)^, which may influence its success^(51–^ ^53^ ^)^ and determine whether it will be adopted or will ‘fit’ the local health system. The four main characteristics relate to the intervention source, evidence strength and quality, relative advantages and adaptability and complexity of intervention^(24)^ (Fig. 2). The intervention source refers its original setting which can affect its adaptability for scaling-up^(35)^ and whether the intervention was piloted internally and externally. Internal testing is considered better^(24)^. Strong, quality evidence supports the implementation of an intervention^(54)^. There are a range of published guidelines or checklists^(55,56)^ that can help researchers assess the evidence quality and strength of an effective intervention.

The relative advantage of one intervention over a similar one is another important characteristic of its ‘effectiveness’^(57)^. For example, an evaluation of a preventive intervention may suggest a relative advantage by finding that ‘reducing a risk factor by a small amount in the overall population is more effective than reducing it by a large amount in high-risk individuals’^(58)^. Assessing existing evidence of the cost of an intervention can help to understand its relative advantages^(51)^. If users perceive a clear, unambiguous advantage in effectiveness or efficiency of the intervention, it is more likely that the implementation will be successful^(24)^.

The fourth characteristic relates to adaptability and complexity. Implementation researchers should aim to understand how an intervention can be adapted, tailored, refined or reinvented to meet local needs^(24)^. This includes considering (i) the essential elements on an intervention and (ii) adaptable elements in the community or the health systems in which it is being implemented^(24)^. Take for example, an intervention to create awareness of a healthy diet in a rural community using text messages on mobile phones. The mobile phone is an essential element and the language of text messages is an adaptable element which may need to be translated into languages used by rural residents. The intervention may not be adaptable if the community is located in a remote location without a mobile network. The complexity relates to perceived difficulties of scaling-up on an intervention. Implementation scientists suggest that complexity could be addressed successfully through simple, clear and detailed implementation plans, schedules and task assignments^(57)^.

### Domain ii: Scaling-up

Under this domain, there are two components: implementation fidelity (Component 2) and course corrections during implementation (Component 3).

#### Component 2: implementation fidelity

**Fig. 3:**
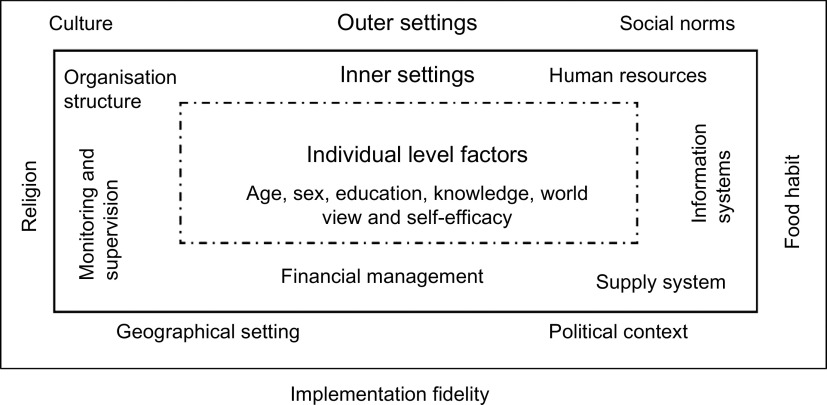
Conceptual framework for implementation fidelity

Fidelity or the degree to which an intervention adheres to the planned process generally produces expected/positive outcomes^(59,60)^ such as effectiveness and population-level benefits. Fidelity is based on three levels of factors adapted from a previous framework^(25)^: (i) outer settings, for example, socio-cultural factors, geographical settings, political context; (ii) inner settings, for example, factors within the organisation or health systems such as organisational policy, structure, human resources, monitoring and supervision and financial management and (iii) individual-level factors such as age, gender, education, skill, worldview, self-efficacy and whether this individual could benefit from or provide an intervention (Fig. 3). The success of scaling-up mostly depends on close consideration of all three levels of contextual factors in tandem with concurrent evaluation and course correction during implementation^(25,61)^. These have been discussed under Components 3 and 5.

The outcomes of an intervention relate to its effectiveness, its economic viability, including cost-effectiveness, and consensus among the stakeholders around scalability of implementation. The effectiveness of the intervention is vital to scaling-up in a real-world setting. Because scaled-up settings often include an entire community, there is limited opportunity to consider a comparison community. Therefore, we proposed a concurrent evaluation (discussed under Component 5). Consideration of economic viability of the intervention, such as the economic capacity of the implementers and a program’s financial sustainability, in this phase is also crucial for its scalability and sustainability^(30,62,63)^. If necessary, researchers can develop an advocacy strategy to encourage the beneficiaries of the intervention to embrace it as well as respective policy-makers and other stakeholders in government departments.

#### Component 3: course corrections during implementation

Assessing the implementation process while it is occurring is crucial to success. Timely course correction creates opportunities for the implementer to revise the implementation plan before it produces an outcome. A concurrent evaluation (discussed under Component 5) allows implementers to assess short-term output/outcomes and implementation gaps. Concurrent evaluations have provided useful lessons for improving the quality of implementation through correction of implementation gaps^(64,65)^. An implementation plan should be flexible enough to include timely course correction, which will be of benefit from the implementers’ perspective and to the beneficiaries and donors^(65)^.

An implementation coordination team may involve key members from the implementing organisation, the funding agency, beneficiary groups and the research organisation. This team should meet regularly to analyse the evaluation data, outcomes, implementation gaps and evaluators’ recommendations^(65)^. Team members should jointly decide how to undertake course correction if required. They might also consider a number of issues including strategies to address the implementation gaps, identification of the individuals to perform course corrections and suggestions to the evaluators about whether any modification is required, for example, adding or dropping the indicators for future assessments of the intervention.

### Domain iii: sustainability

This domain includes the sustainability of an effective intervention and how to assess it. If an evidence-based intervention demonstrated effectiveness and is economically viable when scaled-up, then it is likely to be sustainable in an even larger setting.

#### Component 4: sustainability of interventions and its outcomes

Sustainability should be considered from the beginning of the implementation as it requires planning to define it and to determine the operational indicators to monitor it over time^(47)^. The dimensions of sustainability relate to the amount of investment and inputs required, its spatial coverage over time and its fidelity and adaptability to new real-world contexts^(66)^. The determinants of sustainability mostly relate to programme or intervention-specific factors, organisational and other contextual factors^(66)^. Sustainability is difficult in low-income settings, as it is influenced by economic, political^(47,67)^ and other contextual factors^(68,47,48)^ that are beyond the control of the programme. Reliable funding is critical for maintaining and sustaining the intervention for a long time^(28)^.

Several studies have proposed conceptual frameworks that could be used to assess the sustainability of an intervention^(30,35,47–^ ^51^ ^)^. These frameworks considered sustainability as a standalone component of implementation, whereas we argue that sustainability is closely linked with other components of implementation science. Moreover, the published sustainability frameworks mostly deal with programmes which were implemented in developed country settings. Interventions in low-income settings are generally supported by external funders such as international donor agencies. The determinants, including barriers and opportunities of sustainability, are very context specific in low-income settings. To assess sustainability in low-income settings, it is important to consider following key questions:

1. What would be the appropriate time point to measure sustainability? (e.g. after how many years of implementation, what would be the starting point of implementation, after ending the external supports or initial inception of the intervention?)
2. Would sustainability be measured retrospectively or prospectively?
3. What are the dimensions of sustainability, how would they be measured, should we consider them from the very beginning of intervention innovation?
4. What are the determinants of sustainability?

The answer to these questions will differ depending on the characteristics of the intervention. An implementation scientist may design a conceptual framework including sustainability or may adapt an existing framework taking into account the evidence at the scaling-up phase, the above questions, the implementation setting and the characteristics of the intervention.

#### Component 5: methodological consideration in implementation science

The Component 5 of our framework spans the three domains of our conceptual framework. From the very beginning of implementation research, priority should be given to using an appropriate methodology to identify an ‘effective’ intervention as well as to evaluating it. The proposed framework is likely to be more effective if used in conjunction with a range of appropriate methods suited to each domain.

In Domain i, a review of evidence using need-based formative research, a systematic review or meta-analysis provides a methodologically rigorous approach to systematically synthesising the scientific evidence^(68–^ ^71^ ^)^. Recently, the number of systematic reviews has increased, with an average of eleven systematic reviews of efficacy level trials published every day in various health and nutrition fields^(71)^. Therefore, it is not always necessary to start a comprehensive investigation from the beginning. In some cases, relevant available information could be sufficient to identify the effective intervention. An RCT is considered to be a gold standard experimental research design for evaluating the efficacy or effectiveness of an intervention. However, an RCT might not be always be appropriate because it is not easy to adjust during implementation.

For assessing scalability and sustainability in Domains ii and iii, a concurrent evaluation strategy is used to continuously assess the progress of a particular program in a particular community, to determine how it works and with whom it works and accordingly to make necessary corrections^(15,65)^. This uses multi-methods evaluation – such as qualitative investigations, quantitative assessment, operations research and economic evaluation and may also include other innovative methods to investigate the implementation and its outcome rigorously^(65)^. The scaling-up of an implementation should include a comprehensive evaluation plan containing a range of evaluation methods. For example, the implementation scientist can use process evaluation to assess the implementation process, an impact evaluation to assess outcomes and impact of the implementation and an economic evaluation to assess the cost-effectiveness of the interventions in a real-world setting. Additionally, evaluation at this level should have the provision to generate policy recommendations not only based on the direct outcomes of the interventions but also on the awareness of policy experts about the scalability of the intervention in the wider practice level. Our framework includes a ‘concurrent evaluation’ and considers both process and outcome indicators, addresses short-term and long-term implementation barriers and assesses the determinants and dimensions of sustainability.

### Applying the framework to a real-world nutrition intervention

The above conceptual framework has been tested and validated in a program that distributes MNP widely to address childhood anaemia in Bangladesh. MNP was first developed in the laboratory in 1996 by a group of researchers in the Hospital for Sick Children in Toronto^(72,73).^ Its use was trialled through a series of RCTs conducted in different geographical regions (in seven settings in Ghana, China, Bangladesh and Canada) to assess how effectively it reduced the prevalence of childhood anaemia^(73)^. Following positive results, several trials were then conducted to assess its effectiveness, acceptability and feasibility in small-scale community settings in Bangladesh, Ghana and China^(73)^. In addition, systematic reviews and meta-analyses were used to assess the relevant empirical evidence before recommending that this intervention be scaled-up for use in real-world settings^(74)^. BRAC, formerly known as Bangladesh Rural Advancement Committee – an international development organisation, adopted the intervention to implement a population-based home-fortification program reducing childhood anaemia across Bangladesh. The BRAC model relies on volunteer CHWs^(75)^ to distribute and sell MNP at cost price to carers in their homes who apply it to children’s food.

As noted, BRAC’s CHWs play a critical role in implementing the intervention in communities. Before they were enrolled, it was important to assess their skills, capacity, motivation and willingness – all individual level factors. Similarly, we considered organisational level factors related to BRAC’s implementation process including its use of incentives for CHWs. We have used it to identify key contextual elements such as Bangladesh’s regulations policies, politics and socio-cultural contexts so that the intervention has been successfully scaled-up. The framework is currently being used by the first author and other researchers at icddr,b to guide an ongoing evaluation including whether the program successfully addresses malnutrition, disease prevention or curation and economic feasibility. Empirical evidence of success will create further demand for the intervention among the stakeholders and beneficiaries. Based on that evaluation findings and experiences, the following papers are under review:

1. Home Fortification of Food with Micronutrient Powder: Critical Considerations to Implement It in a Real-World Setting
2. Factors Associated with Home-visits by Volunteer Community Health Workers to Implement a Home-fortification Intervention in Bangladesh: A Multilevel Analysis
3. Performance of Volunteer Community Health Workers in Implementing Home-fortification Interventions in Bangladesh: A Qualitative Investigation
4. Role of Home-visit by Volunteer Community Health Workers in Implementation of Home-fortification of Foods with Micronutrient Powder in Rural Bangladesh
5. Use of Concurrent Evaluation to Improve Implementation of a Home-fortification Programme in Bangladesh: A Methodological Innovation
6. Cost-effectiveness of a Market-Based Home-fortification of Food with Micronutrient Powder Programme in Bangladesh

In nutrition implementation science, choosing an appropriate methodology is another critical task^(68)^. Scholars propose a range of methodologies to evaluate an implementation^(76)^, or to measure the outcomes or impacts^(77)^ in general but they rarely focus specifically on the most appropriate methods for identifying, testing and improving an innovative nutrition intervention. When a nutrition intervention is implemented in a real-world setting, appropriate methodology is required to carefully assess the contexts, process, outcome, cost and effectiveness of the implementation. Our framework proposes concurrent evalution with a range of appropriate methods proposed for each phase, that is, to identify an effective intervention; explore barriers and facilitators; address gaps during implementation and to show a pathway to sustain the implementation and its outcomes over time.

## Discussion

Our framework offers some advances on existing frameworks in implementation sciences. It specifically captures all the main elements of implementation science. This has created an opportunity to understand implementation science holistically and comprehensively – from the beginning to the end including identifying an effective intervention, understanding the process of implementation and effectiveness and finally the sustainability of intervention. Combining the three domains will help implementation researchers to visualise the whole process of implementation science in a one frame.

A unique characteristic of our framework is the inclusion of course correction during implementation. In the implementation literature, most papers focus on how implementation fidelity could be achieved^(27,42,50,60)^. However, few reviewed frameworks considered how to make course corrections. Concurrent evaluation may generate evidence around the implementation process that is adjustable and the short-term and long-term outcomes. This has improved implementation fidelity and coverage of a home-fortification intervention in Bangladesh^(65)^. The combination of concurrent evaluation and course correction creates an opportunity to improve implementation fidelity as well as reducing the uncertainty, which ultimately contributes to implementation sustainability.

Sustainability of an intervention and its outcomes are another key implementation science concern. Issues related to sustainability are disruption and discontinuous financial supports, poor support from local stakeholders and local implementers’ lack of decision-making capacity. During decision making, local authorities commonly question why they should invest in the intervention, how they should invest, what are the expected challenges and outcomes. Our framework is particularly helpful for answering these questions, for engaging key stakeholders and generating shared lessons learned through documenting a complete pathway of implementation.

This framework also shows how an intervention can be made more sustainable and how it may be assessed. It is important that sustainability should be considered from the beginning^(48)^ of the intervention because when an effective intervention is scaled-up, it is often changed to adapt within new real-world contexts. Such adaptation ultimately strengthens implementation fidelity so that it is flexible enough to cope with contextual barriers and facilitators. Once the scaled-up intervention generates effective outcomes, then it is more likely to be sustained within that real-world setting.

The framework is holistic. However, this raises the question of whether a single project can implement such a broad framework. Our argument is that it is possible. For example, a single project may not have to start with innovative scientific evidence generation, if a proven effective intervention already exists. If this is the case, the project should follow the pathway for how the intervention can be scaled-up and made sustainable.

### Limitations

The proposed framework was developed based on a review of peer-reviewed journal articles and may have excluded useful frameworks available elsewhere such as the grey literature. Most of the reviewed frameworks were originally developed for non-nutrition fields as there is limited literature on the application of implementation science to nutrition interventions. Our framework also derives, in part, from the first author’s hands-on experience in implementing and evaluating a nutrition intervention in Bangladesh. This informed the development of the framework and methodological considerations.

### Conclusions

This review provides a foundational and holistic understanding of key components of a conceptual framework and aims to make a valuable addition to implementation science. The framework provides guidance for implementation researchers, policy-makers and programme managers on: reviewing key characteristics of an effective intervention, opportunities to strengthen the fidelity and outcomes, and on sustainability. It may be used in many fields but it aims to assist the development of nutrition interventions for use, particularly in low-income settings taking into account the contexts and characteristics of the intervention. Researchers can use it to plan an implementation roadmap and as a tool for negotiating with funders and other key stakeholders. We encourage future researchers to use and validate this conceptual framework for other public health interventions in various settings and propose improvements.
